# Navigating Nutritional Inequality in Schizophrenia: A Comprehensive Exploration of Diet, Genetics, and Holistic Management Across the Life Cycle

**DOI:** 10.3390/nu16213738

**Published:** 2024-10-31

**Authors:** Yiming Yan, Disheng Zhou, Jianhua Chen

**Affiliations:** 1 Shanghai Mental Health Center, Shanghai Jiao Tong University School of Medicine, Shanghai 200030, China; ym.yan@sjtu.edu.cn; 2 Shanghai Clinical Research Center for Mental Health, Shanghai Key Laboratory of Psychotic Disorders, Shanghai Jiao Tong University School of Medicine, Shanghai 200030, China; 3 School of Nursing, Li Ka Shing Faculty of Medicine, The University of Hong Kong, Hong Kong SAR, China; zdison@connect.hku.hk

**Keywords:** nutritional inequality, schizophrenia, nutrigenetics, nutrigenomics, multi-omics

## Abstract

This review explores the understudied topic of nutritional inequality among individuals with schizophrenia, highlighting the complex interplay between diet, genetics, and mental health. Unhealthy dietary patterns, socioeconomic factors, and disordered eating behaviors contribute to malnutrition, increasing the risk of physical health issues and premature mortality. Socioeconomic factors exacerbate nutritional disparities, necessitating targeted interventions. Genetic influences on nutrient metabolism remain under-researched, although nutritional genomics shows potential for personalized interventions. Current research reveals methodological gaps, urging larger sample sizes and standardized approaches. The integration of nutrigenomics, encompassing various omics disciplines, emerges as a transformative tool. The holistic life-cycle approach to schizophrenia management underscores the vital role of nutrition, calling for personalized interventions to enhance mental health outcomes.

## 1. Introduction

People with schizophrenia experience a significantly shorter lifespan compared to the general population, with a difference of 14.5 years [1]. This premature mortality is largely attributed to physical health conditions such as cardiovascular diseases and metabolic disorders [2]. The United Nations’ Sustainable Development Goal (SDG) target 3.4 emphasizes the importance of reducing premature mortality from non-communicable diseases. Therefore, it is crucial to study the comprehensive epidemiological landscape of premature mortality in individuals with schizophrenia in order to understand the underlying mechanisms and establish a fundamental framework in those suffering from this condition.

Growing research in the general population have shown that adopting healthy dietary patterns with a scientifically balanced nutritional structure may decrease the morbidity rates of certain diseases, including cardiovascular and metabolic diseases [3,4]. Conversely, an unhealthy diet and malnutrition may raise the risk of corresponding diseases [5,6]. This evidence highlights the significance of diet and nutrition in overall health and suggests that it can be a population-level strategy to address premature mortality from all causes, including among individuals with schizophrenia.

Although there is a general recognition that individuals with schizophrenia lead unhealthy lifestyles, especially in terms of their diet [7,8], there has been surprisingly limited research dedicated to this critical aspect. Nutritional inequality may refer to the discrepancies in the quality, consumption, and utilization of nutrients among different population groups [9]. This concept encompasses various factors, including socioeconomic status, cultural practices, systemic barriers, and biological differences that contribute to an uneven distribution of health-promoting diets and, consequently, differences in nutritional status.

This narrative review investigates the presence of nutritional disparities among individuals with schizophrenia, the potential consequences of these disparities for their quality of life, and possible strategies for enhancing their overall health. It highlights a rather important but under-investigated area that needs further research.

## 2. Dietary and Nutritional Condition in Individuals with Schizophrenia

Individuals with schizophrenia tend to follow an unhealthy dietary pattern, characterized by a higher intake of fat, especially saturated fat, and a lower consumption of fiber [7,10]. A Korean study revealed that male patients diagnosed with schizophrenia had significantly reduced dietary intake of protein, polyunsaturated fatty acids (PUFAs), vitamin K, niacin, folate, and vitamin C compared to males in the control group [11]. To provide more accurate information, a recent meta-analysis pointed out that individuals with schizophrenia consumed an additional +1695 kJ of energy per day compared to controls [12]. Meanwhile, several studies have emphasized unhealthy dietary habits among individuals with schizophrenia, including fast eating and inadequate water intake per day [13,14]. Furthermore, a group of Egyptian patients diagnosed with schizophrenia exhibited higher levels of disordered eating attitudes, as assessed by the EAT40, compared to the control group [15], which may further contribute to imbalanced nutritional intake. Another important dietary distortion is the prevalence of alcohol use in individuals with schizophrenia. A systematic review and meta-analysis points out that 24.3% of the patients are likely to have an alcohol disorder in their lifetime [16], which is much higher than in the general public (11% in U.S. [17]). Meanwhile, consumption of alcohol can impair the absorption of essential nutrients [18,19], potentially exacerbating nutritional deficiencies that are already common in individuals with schizophrenia.

Individuals with schizophrenia also exhibit differences in nutritional utilization compared to the general population, which adds complexity to the condition. It has been suggested that people with schizophrenia might have distorted one-carbon metabolism, which primarily deals with the transfer of methyl groups and is crucial for amino acid metabolism, nucleotide synthesis, neurotransmitter synthesis, and methylation reactions [20,21]. Insufficient intake or absorption of folate and vitamin B12 can further impair one-carbon metabolism, leading to disruptions in nucleotide synthesis, increased homocysteine levels [20], and potential health issues, such as cardiovascular diseases [22]. Additionally, evidence indicates that the tryptophan/kynurenine pathway, which is involved in the metabolism of tryptophan, appears to be abnormal in individuals with schizophrenia, influenced by factors such as inflammation, stress, genetic variants, and potentially brain-specific immune processes [23]. Both of these metabolic pathways involve amino acids and other nutrients that are easily available in daily foods, implying the complex and important role of nutrition-related pathways in individuals with schizophrenia.

Overall, the inappropriate intake of macronutrients and micronutrients, distorted dietary habits and attitudes, and disordered nutritional utilization are three topics that deserve attention in the context of individuals with schizophrenia.

The WHO describes malnutrition as deficiencies or excesses in nutrient intake, imbalances of essential nutrients, or impaired nutrient utilization, encompassing both undernutrition and overweight/obesity, as well as diet-related noncommunicable diseases. Therefore, the unbalanced dietary and nutritional structure observed in individuals with schizophrenia can be considered a form of malnutrition, highlighting the presence of structural nutritional inequality. This nutritional inequality demands urgent investigation and swift action to address its profound implications [24].

In the general population, evidence supports the notion that malnutrition is a key contributor to physical disorders or diseases in various systems, including the cardiovascular, metabolic, and immune systems, etc. [25,26,27]. Clinical experiences and research findings have consistently shown that premature death in individuals with schizophrenia might be largely attribute to physical diseases affecting the cardiovascular and metabolic systems, as mentioned above. Unfortunately, there is a limited number of studies investigating how malnutrition may trigger physical diseases and impact mental conditions such as relapse. Therefore, it is urgent to study the underlying structure of nutritional inequality in individuals with schizophrenia and take action to alleviate the heavy burden of premature mortality.

## 3. Socioeconomic Factors in Nutritional Inequality Reduce Nutrition Quality in People with Schizophrenia

Socioeconomic factors have a significant impact on the nutritional situation of individuals with schizophrenia, resulting in nutritional inequality. People with schizophrenia often face unique challenges related to their socioeconomic status, which can affect their access to quality nutrition and overall dietary well-being. Studies in the general population have pointed out the complex relationship between socioeconomic status and nutritional equality, particularly highlighting the heavy burden of malnutrition in low- and middle-income countries, especially within the poorest income quartile [28].

Schizophrenia can impact an individual’s ability to maintain employment [29] due to symptoms such as cognitive impairments and social withdrawal. This can result in unemployment or underemployment, leading to financial difficulties for individuals with schizophrenia. Consequently, many individuals may experience financial constraints [30], which can affect their ability to afford a balanced and nutritious diet. The cost of healthy food options, such as fresh fruits and vegetables, can be a barrier for those with limited financial means. Additionally, industrialized food systems often provide ultra-processed foods, which are high in calories but low in nutritional value, at a lower price [31]. This may become the primary dietary choice for individuals with lower socioeconomic status. Furthermore, some individuals with schizophrenia may experience housing instability or even homelessness [29,32], which can further complicate their ability to store and prepare nutritious meals. These socioeconomic factors contribute to the nutritional inequality observed in people with schizophrenia.

Individuals with schizophrenia may also face challenges in accessing education and acquiring knowledge about nutrition to improve their health [33]. Limited education can impact their understanding of the importance of a balanced diet and how to make informed food choices. Additionally, social withdrawal due to the negative symptoms of schizophrenia [34] may reduce their access to social networks that may provide support in terms of meal preparation, grocery shopping, and sharing nutritional knowledge. Social support is crucial for maintaining healthy dietary habits.

More studies are required to investigate how socioeconomic factors lead to nutritional inequality in individuals with schizophrenia. There is a limited number of studies focusing on individuals with schizophrenia compared to the general population, which also represents inequality.

## 4. Disordered Eating Behavior in Schizophrenia Influencing the Consumption of Nutrients Causes Nutritional Inequality

Eating behaviors in individuals with schizophrenia are complicated, and disordered eating behaviors are not uncommon in this population. Individuals with schizophrenia may have disrupted eating habits and attitudes [14,15], as mentioned above, while some may experience pathological disordered eating with varying severity and manifestations.

Antipsychotic medications, which are the cornerstone of symptom management in schizophrenia, can have side effects on metabolism, appetite, and weight [35,36,37,38,39,40]. Many current antipsychotics, such as olanzapine and clozapine, tend to stimulate appetite, leading to abnormal weight gain and an increased risk of type 2 diabetes and cardiovascular disease in individuals with schizophrenia [41]. Consequently, antipsychotic medications may contribute to overconsumption in some individuals with schizophrenia, resulting in an energy surplus and an unbalanced dietary structure.

A literature review suggests that there is a comorbidity between schizophrenia and eating disorders, with a prevalence of approximately 10% in binge eating disorder and night eating syndrome, and 1–4% in anorexia nervosa [42]. These eating disorders may or may not be related to psychotic symptoms [42], including a distorted body image. Additionally, individuals with schizophrenia often have other comorbid mental conditions, such as depression or anxiety [43,44], which can impact eating habits, leading to emotional eating [45].

The executive function and cognitive impairments in schizophrenia [46] may impact various aspects of daily living, including meal planning and nutritional decision-making. Negative symptoms of schizophrenia, such as apathy, social withdrawal, and reduced motivation, can result in decreased interest in self-care activities [47,48]. It is important to note that swallowing problems in individuals with schizophrenia may be life-threatening [49]. All of these factors can contribute to irregular eating patterns and nutritional deficiencies. Furthermore, stigmatization and social isolation associated with schizophrenia may also cause emotional distress [50], which partly contributes to the avoidance of social situations involving food and the development of disordered eating behaviors.

Overall, antipsychotic medications, eating-related comorbidities, and physical and mental impairments can induce nutritional inequality and malnutrition in patients with schizophrenia. This may explain the high prevalence of comorbid physical diseases among this population. Therefore, gaining a better understanding of the mechanisms underlying nutritional inequality in people with schizophrenia may be a powerful approach to addressing the significant burden of physical diseases and potentially improving their mental well-being.

## 5. Current Nutritional Research in Therapeutic Strategies of Schizophrenia

Nowadays, more studies are shifting to focus on the role of diet-related interventions in the treatment of schizophrenia. These interventions can be explored through two possible pathways: (1) Diet can be used in conjunction with psychiatric medications to enhance the treatment of schizophrenia, and (2) diet may help reduce the side effects associated with psychiatric drugs (Table 1). The side effects associated with psychiatric drugs can be wide-ranging and often impact both physical and mental health. Commonly prescribed antipsychotic medications, for instance, are linked to metabolic side effects such as weight gain, increased blood glucose levels, insulin resistance, and dyslipidemia, which elevate the risk of developing conditions such as obesity, type 2 diabetes, and cardiovascular disease [51,52]. By tailoring dietary interventions to address these drug-induced side effects, clinicians can help support overall health and improve outcomes for individuals undergoing psychiatric treatment.

As mentioned earlier, individuals with schizophrenia tend to have an unhealthy diet characterized by higher saturated fat intake and lower levels of essential fatty acids (EFAs). The deficiency of specific EFAs may hinder normal nerve cell-membrane metabolism. Therefore, supplementing omega-3 fatty acids may enhance the effectiveness of conventional treatment for schizophrenia. In a study, participants who received a combination of original antipsychotic treatment and omega-3 fatty acid supplementation for 8 weeks experienced a significant decrease in schizophrenia symptoms compared to those who received only conventional antipsychotics [53]. In a review, the authors summarized how omega-3 fatty acids showed potential in reducing the risk of psychosis in ultra-high-risk patients and first-episode psychosis [54]. Although the number of RCTs included in this review is limited (one multicenter RCT study for ultra-high-risk patients and two RCT studies for first-episode psychosis), it still shows there may be promising strategies considering omega-3 fatty acids to combat schizophrenia in an early phase. Similarly, the supplementation of vitamin D and probiotics has shown significant beneficial effects on the severity of symptoms measured by the Positive and Negative Syndrome Scale (PANSS) score in patients with schizophrenia [55]. The PANSS is commonly used to assess symptom severity in schizophrenia. The intake of dietary amino acids has also been noted since the last century, but researchers have yet to reach a unanimous conclusion on this topic. Tryptophan, in particular, has garnered attention as a precursor to serotonin, a neurotransmitter implicated in schizophrenia, since the serotonin hypothesis was put forward in the 1970s [56,57]. A study in the 1990s indicated that a low-tryptophan diet might be effective as an adjuvant treatment for schizophrenia [58]. However, due to limited research methods, a deeper understanding of how tryptophan acts on the human body is still lacking. Recent research has shown that acute oral intake of tryptophan has a different effect on cognition and cerebral blood flow in individuals with schizophrenia and healthy controls [59]. More randomized controlled trials are expected in this area.

Some researchers have concentrated on improving the imbalanced dietary patterns of schizophrenia patients, but with limited success. In one study, free fruit and vegetables were provided to schizophrenia patients for 6 weeks because of their low intake of these foods in their original diet. The results indicated that participants had good compliance during the intervention. However, 12 months later, they reverted to their original eating pattern [60]. This suggests that individuals with schizophrenia may face challenges in adopting new dietary patterns due to executive function deficits, motivational deficits, and socioeconomic factors, leading to difficulties in maintaining adherence. Further research is needed to explore more effective approaches to help people with schizophrenia improve their dietary patterns.

Antipsychotic medications used to treat schizophrenia are known to have side effects, such as abnormal weight gain and metabolic disturbances [61], which can increase the risk of premature death from conditions such as diabetes and cardiovascular disease. Combining these medications with specific dietary interventions may offer a potential solution to mitigate these concerns. For example, a 12-week supplementation of probiotics and dietary fiber has been found to significantly reduce weight gain induced by olanzapine, a commonly used second-generation antipsychotic in schizophrenia patients [62]. Additionally, the supplementation of vitamin D and probiotics for 12 weeks has been proven to improve glucose and lipid metabolism by reducing fasting glucose, insulin resistance, triglycerides, and total cholesterol levels [55]. Some researchers have proposed orthomolecular medicine as an alternative approach to traditional drugs for schizophrenia. This approach involves providing high doses of vitamins to regulate vitamin levels in the body. While early studies showed some promising results with niacin [63] and ascorbic acid [64], nutritionists argue that orthomolecular medicine lacks sufficient evidence. A 5-month megavitamin treatment with vitamins A, B, C, D, and E was found to increase serum vitamins levels, but no positive effect was observed in treating schizophrenia [65].

In conclusion, the current state of scientific research on nutritional therapeutic strategies for schizophrenia reveals notable gaps and limitations. The existing knowledge is hindered by small sample sizes, inconsistent methodologies, and an overreliance on observational studies. Despite recognizing the impact of nutrition on mental health, the available literature on nutritional interventions specifically tailored for individuals with schizophrenia remains insufficient. There is a marked absence of comprehensive methodologies that explore the intricate relationship between various nutrients and the complex symptomatology of schizophrenia. Future research in the field of nutritional interventions for schizophrenia should aim to address these gaps in methodologies and outcomes by utilizing emerging cutting-edge technologies to advance our understanding and improve the effectiveness of therapeutic nutritional approaches for individuals with schizophrenia.

**Table 1:** Examples of current research on nutritional therapeutic strategies for schizophrenia. Studies are often limited by small sample sizes, inconsistent methodologies, and an overreliance on observational data, which hinder the development of effective interventions. Although the impact of nutrition on mental health is acknowledged, there remains a shortage of comprehensive studies exploring the detailed effects of specific nutrients on the complex symptoms of schizophrenia. Future research should address these gaps by adopting innovative technologies and rigorous methodologies to better understand and enhance the therapeutic potential of nutrition for schizophrenia treatment.

Paper	Intervention	Control	Duration	Reported Effects Compared to Control
Jamilian et al., 2014 [53]	Original antipsychotic treatment plus Omega-3 fatty acids	Original antipsychotic treatment plus placebo	8 weeks	Significant decrease in symptoms of schizophrenia
Ghaderi et al., 2019 [55]	Vitamins plus probiotics	Placebo	12 weeks	Significant improvement in the general and total Positive and Negative Syndrome Scale (PANSS) scores; Significant improvement in fasting glucose, insulin resistance, fasting total cholesterol, and triglyceride
Rosse et al., 1992 [58]	Tryptophan (TRP)-deficient diet	NA	4 days	Minimal improvements in objective ratings of the severity of psychotic symptomatology
Hare et al., 2023 [59]	A single oral dose of tryptophan (6 g)	Placebo	Acute	No significant effect on cognitive assessment; significant increase in cerebral blood flow in healthy controls but not in people with schizophrenia
Huang et al., 2022 [62]	Olanzapine plus probiotics	Olanzapine	12 weeks	No significant difference in weight gain; significant decrease in insulin resistance index (IRI)
Huang et al., 2022 [62]	Olanzapine plus probiotics and dietary fiber	Olanzapine	12 weeks	Significantly lower weight gain and IRI increase induced by olanzapine
Vaughan et al., 1999 [65]	Megavitamin treatment with vitamins A, B, C, D, E	25 mg of vitamin C	5 months	No consistent self-reported symptomatic or behavioral differences
McCreadie et al., 2005 [60]	Provided free fruit and vegetables	Usual diet	6 months	Significant increase in fruit and vegetable intake during intervention while consumption fell to pre-intervention levels 12 months after intervention

## 6. Nutrigenetics of Schizophrenia: An Under-Researched Territory

It has long been recognized that people react differently to macronutrients and micronutrients, and this can be partly attributed to genetic factors [66]. Nutrigenetics is a field of study that investigates how genetic variation, especially single-nucleotide polymorphisms (SNPs), contributes to an individual’s various responses to nutrients [67]. For example, the cholesteryl ester transfer protein (*CETP*) gene may influence an individual’s level of postprandial lipemia [68]. Similarly, SNPs in the vitamin D receptor (*VDR*) gene have been found to impact the response to vitamin D supplements [69], and genetic polymorphisms in the cytochrome P450 1A2 (*CYP1A2*) gene have been considered to be associated with an individual’s response to caffeine [70]. Furthermore, evidence suggests that serum caffeine and its metabolites may serve as biomarkers of Parkinson’s disease [71,72], indicating a complex interaction between nutrients and diseases. Therefore, we can understand that there is a complex interplay between nutrition and genes, which profoundly influences how individuals respond to nutrients based on their genetic makeup.

Considering that schizophrenia is a complex genetic disorder involving hundreds of genes [73], it is reasonable to speculate that people with schizophrenia may exhibit specific biological profiles in nutrient utilization. Polygenic scores for schizophrenia have been found to be strongly associated with nutrient intake, including carbohydrates, fats, proteins, calcium, carotene, folate, iron, and vitamins B6, B12, D, and E [74]. This indicates the existence of a common biological pathway linking nutrition and schizophrenia. However, further research is needed to determine how individuals with schizophrenia may respond differently to specific nutrients and to draw reliable conclusions. This area of research is referred to as “nutrigenetics”, which aims to explore how an individual’s genetic profile influences their phenotypic response to nutritional intake [67]. While nutrigenetics has made significant progress in non-communicable diseases such as obesity and cardiovascular diseases [66,75], there is still a lack of exploration in the nutrigenetics of mental disorders such as schizophrenia.

The current research area on nutrition in schizophrenia focuses on whether specific nutrients may be pathogenic or therapeutic. However, the existing research evidence in this field, accumulated since the last century, is mixed and needs further integration and standardization. To gain a better understanding of the nutrient utilization profile in schizophrenia, future research can explore the genetic basis of distorted one-carbon metabolism and the tryptophan/kynurenine pathway mentioned above. An analysis of the PGC-SCZ dataset revealed an interaction in a locus containing indoleamine 2,3-dioxygenase 2 (*IDO2*), which is involved in the tryptophan/kynurenine pathway and has immunoregulatory functions. This interaction was found to have a sex-dependent effect on the risk of schizophrenia and plausibly due to different kynurenic acid expression between the sexes [76]. Another important review pointed out the critical biological role of the tryptophan/kynurenine pathway in schizophrenia [77]. The underlying mechanism suggests that peripheral nutritional and inflammatory signals may lead to the accumulation of kynurenines in neural systems, contributing to mental disorders. However, engaging in physical activities that eliminate kynurenine could have an ameliorating effect. In summary, it can be inferred that the genetic background of schizophrenia influences the metabolism of specific nutrients (e.g., amino acids), which, in turn, contributes to the pathogenesis (schizophrenia risks). However, the accumulation of metabolites can be reversed through the clearance of these metabolites via physical activity. The evidence mentioned above provides us with a perspective on how the interaction between genes, nutrients, and disease may play a pivotal role in schizophrenia and other diseases. This suggests that nutrigenetics can be a promising approach to study the pathogenesis and treatment of schizophrenia.

Another important area, as discussed above, is the impact of alcohol consumption on individuals with schizophrenia. Examining this effect in the context of genetic predispositions could offer valuable insights, as genetic factors may influence both susceptibility to alcohol-related nutrient deficiencies and individuals’ nutritional needs. Including this discussion would contribute to a more comprehensive understanding of how genetic information can inform personalized nutrition approaches for managing schizophrenia and its common comorbidities, thereby supporting more tailored and effective therapeutic strategies.

## 7. Nutritional Implications of Gene-Nutrition Interaction in Schizophrenia: Where Nutrigenomics May Kick In

Nutritional genomics, which combines nutrigenetics and nutrigenomics, is a field of scientific research that investigates the interaction between an individual’s genetic makeup (nutrigenetics) and their responses to nutrients and dietary components [67,75]. By studying specific genes, nutritional genomics aims to understand how genetic variations impact nutrient digestion, absorption, and metabolism, ultimately contributing to variations in health outcomes. This research helps to better understand the complicated interaction between genetics and nutrients in individuals with schizophrenia.

Nutrigenomics is a broader field that encompasses the study of how nutrients impact multiple phenotypes, which are represented as multi-omics such as genomics, epigenomics, transcriptomics, proteomics, metabolomics, lipidomics, glycomics, and microbiomics [66,67,78]. Ongoing technological developments in translating and integrating these multi-omics codes offer hope for developing an overall understanding of nutritional inequality in individuals with schizophrenia. These new technologies provide a brand-new perspective for examining therapeutic strategies for schizophrenia, which has been characterized by a prolonged history of modest advancements. They hold the promise of transformative change and offer a potential breakthrough that has long eluded the field.

As discussed above, nutrigenetics can influence an individual’s responses to specific nutrients. By studying the genomics of individuals with schizophrenia from a nutritional perspective, we can identify genetic variations associated with nutritional inequalities and develop tailored dietary and nutritional interventions [79]. Transcriptomic and proteomic studies help identify changes in gene expression in response to dietary interventions [80]. Additionally, metabolomics and lipidomics further explore how the human body responds to dietary interventions [75,80], which can have therapeutic effects on individuals with schizophrenia. Several researches have focused on the metabolomics of the tryptophan/kynurenine pathway in people with schizophrenia [77,81,82], indicating the potential of metabolites of tryptophan as biomarkers in schizophrenia management. However, the complicated interaction within the multi-omics of the tryptophan/kynurenine pathway still requires further study, as it may elucidate undiscovered mechanisms and therapeutics for schizophrenia.

Epigenomics uncovers the intricate interplay between nutrition and gene regulation [83], shedding light on the vital role of DNA methylation, histone modifications, and non-coding RNAs as crucial epigenetic mechanisms influenced by dietary factors [84,85]. In individuals with schizophrenia, abnormal epigenetic patterns may lead to the disorder, and nutritional interventions are expected to modulate these patterns [86]. Understanding how nutrition affects the epigenetic landscape in schizophrenia allows for the identification of specific targets and the customization of interventions based on individual epigenetic profiles, thereby facilitating the development of precise and effective nutritional strategies. Simultaneously, the gut microbiome plays a crucial role in nutrient metabolism and can influence brain function through the gut-brain axis [87]. Microbiomic studies can reveal the interplay between nutrients and gut microbiota, revealing complex and significant relationships, such as the impact of kynurenine metabolism and gut microbiota on overall health [77]. These interactions have implications for the mental and physical well-being of individuals with schizophrenia.

However, the integration of cutting-edge nutrigenomics technologies into the investigation of nutritional disparities in schizophrenia, which has the potential to unravel personalized dietary responses based on genetic makeup, is noticeably lacking in current research. This dearth of investigation hampers the development of targeted and personalized nutritional interventions that could significantly contribute to the holistic management of schizophrenia.

## 8. Holistic Schizophrenia Prevention and Management Across the Life Cycle: A Nutritional Perspective

The clinical courses of schizophrenia may be concluded as (1) prenatal exposure to adversity such as perinatal complications; (2) childhood adversity; (3) prodrome; (4) first-episode psychosis; (5) treatment; (6) relapse, and (7) chronic phase [34]. Managing this mental health condition throughout the entire life cycle is crucial, with a particular emphasis on nutrition. Schizophrenia is a chronic and complex disorder that evolves over time, and nutrition plays a multifaceted role in mental health. A comprehensive approach that spans the entire life cycle, starting from prenatal development to old age, recognizes the dynamic nature of schizophrenia and the significant impact of nutrition on mental well-being.

Adequate prenatal nutrition is particularly important for fetal brain development [88]. Studies on the Dutch Hunger Winter of 1944–1945 [89] and the Great Chinese Famine of 1959–1961 [90] have shown that prenatal malnutrition increases the risk of schizophrenia later in life. To better understand the underlying mechanisms of how nutrients influence schizophrenia pathogenesis, it is essential to conduct research using large birth cohorts that consider prenatal conditions. The Avon Longitudinal Study of Parents and Children (ALSPAC) [91] is an example of such a study that can provide valuable insights from a nutrigenomic perspective. By optimizing maternal nutrition, including sufficient intake of folic acid, iron, and other essential nutrients [92,93], it may be possible to reduce the risk of schizophrenia outcomes.

Early detection and intervention during the onset of schizophrenia are crucial for better outcomes [94]. While nutritional support accompanied by standard treatments may contribute to overall mental and physical well-being, the evidence is still insufficient to draw a definitive conclusion. It is also important to address drug-food interactions with careful consideration of genomics to optimize treatment outcomes and minimize side effects [95]. Antipsychotic medications, often prescribed for long-term management, can have adverse effects such as constipation [96], which has an impact on quality of life and treatment compliance. Dietary intervention may be useful in addressing these effects. As individuals with schizophrenia age, addressing their nutritional needs becomes increasingly important. Providing proper support for nutrition in older adults with chronic schizophrenia helps reduce chronic conditions and maintain cognitive health and overall well-being [97,98] in the later stages of life. However, there is a lack of evidence on these topics, and further nutrigenomic research is needed.

In essence, taking a life-cycle approach to schizophrenia management emphasizes the importance of addressing nutritional needs at various stages to enhance overall mental health outcomes. Nutrition plays a vital role in the mind-body connection, and a holistic approach to mental health considers the impact of diet on mood, cognitive function, and overall well-being. An individualized nutritional plan that takes into account factors such as genetics, medication interactions, coexisting health conditions, and personal preferences can be integrated into the continuum of care to enhance the effectiveness of nutritional interventions for individuals with schizophrenia.

## 9. Conclusions

In conclusion, this review emphasizes the critical yet under-explored area of nutritional inequality in individuals with schizophrenia. The complex interplay between diet, genetics, and the pathophysiology of schizophrenia raises concerns about the heightened risk of physical health issues and premature mortality in this population. Unhealthy dietary patterns, distorted eating behaviors, and impaired nutrient utilization contribute to malnutrition, emphasizing the urgent need for comprehensive investigation and intervention. Current nutritional research in therapeutic strategies reveals gaps and limitations, with insufficient methodological rigor and an overreliance on observational studies. It is important to stress the need for larger sample sizes and standardized methodologies, as well as the integration of emerging technologies, such as nutritional genomics, to enhance our understanding of the complex interactions between genetics, nutrition, and schizophrenia. The lack of investigation in nutritional genomics hinders the development of personalized nutritional interventions that could significantly contribute to the holistic management of schizophrenia. Taking a holistic approach to schizophrenia prevention and management across the life cycle underscores the importance of nutrition. Recognizing the dynamic nature of schizophrenia from prenatal development to old age and addressing nutritional needs at various stages can enhance overall mental health outcomes. Integrating nutrition into the continuum of care by considering genetics, medication interactions, and individual preferences offers a tailored and effective approach.

## References

[1] Hjorthøj C., Stürup A.E., McGrath J.J., Nordentoft M. (2017). Years of potential life lost and life expectancy in schizophrenia: A systematic review and meta-analysis. Lancet Psychiatry.

[2] Correll C.U., Solmi M., Croatto G., Schneider L.K., Rohani-Montez S.C., Fairley L., Smith N., Bitter I., Gorwood P., Taipale H. (2022). Mortality in people with schizophrenia: A systematic review and meta-analysis of relative risk and aggravating or attenuating factors. World Psychiatry.

[3] Jayanama K., Theou O., Godin J., Cahill L., Shivappa N., Hébert J.R., Wirth M.D., Park Y.M., Fung T.T., Rockwood K. (2021). Relationship between diet quality scores and the risk of frailty and mortality in adults across a wide age spectrum. BMC Med..

[4] Naghshi S., Sadeghi O., Willett W.C., Esmaillzadeh A. (2020). Dietary intake of total, animal, and plant proteins and risk of all cause, cardiovascular, and cancer mortality: Systematic review and dose-response meta-analysis of prospective cohort studies. BMJ.

[5] Chien S.C., Chandramouli C., Lo C.I., Lin C.F., Sung K.T., Huang W.H., Lai Y.H., Yun C.H., Su C.H., Yeh H.I. (2021). Associations of obesity and malnutrition with cardiac remodeling and cardiovascular outcomes in Asian adults: A cohort study. PLoS Med..

[6] Kazemi A., Sasani N., Mokhtari Z., Keshtkar A., Babajafari S., Poustchi H., Hashemian M., Malekzadeh R. (2022). Comparing the risk of cardiovascular diseases and all-cause mortality in four lifestyles with a combination of high/low physical activity and healthy/unhealthy diet: A prospective cohort study. Int. J. Behav. Nutr. Phys. Act..

[7] Brown S., Birtwistle J., Roe L., Thompson C. (1999). The unhealthy lifestyle of people with schizophrenia. Psychol. Med..

[8] van Zonneveld S.M., Haarman B.C.M., van den Oever E.J., Nuninga J.O., Sommer I.E.C. (2022). Unhealthy diet in schizophrenia spectrum disorders. Curr. Opin. Psychiatry.

[9] Nisbett N. (2019). Understanding the nourishment of bodies at the centre of food and health systems–systemic, bodily and new materialist perspectives on nutritional inequity. Soc. Sci. Med..

[10] Dipasquale S., Pariante C.M., Dazzan P., Aguglia E., McGuire P., Mondelli V. (2013). The dietary pattern of patients with schizophrenia: A systematic review. J. Psychiatr. Res..

[11] Kim E.J., Lim S.Y., Lee H.J., Lee J.Y., Choi S., Kim S.Y., Kim J.M., Shin I.S., Yoon J.S., Yang S.J. (2017). Low dietary intake of n-3 fatty acids, niacin, folate, and vitamin C in Korean patients with schizophrenia and the development of dietary guidelines for schizophrenia. Nutr. Res..

[12] Teasdale S.B., Ward P.B., Samaras K., Firth J., Stubbs B., Tripodi E., Burrows T.L. (2019). Dietary intake of people with severe mental illness: Systematic review and meta-analysis. Br. J. Psychiatry.

[13] Simonelli-Muñoz A.J., Fortea M.I., Salorio P., Gallego-Gomez J.I., Sánchez-Bautista S., Balanza S. (2012). Dietary habits of patients with schizophrenia: A self-reported questionnaire survey. Int. J. Ment. Health Nurs..

[14] Zurrón Madera P., Casaprima Suárez S., Álvarez L.G., García-Portilla González M.P., Junquera Fernández R., Lluch Canut M.T. (2022). Eating and nutritional habits in patients with schizophrenia. Rev. Psiquiatr. Salud Ment. (Engl. Ed.).

[15] Fawzi M.H., Fawzi M.M. (2012). Disordered eating attitudes in Egyptian antipsychotic naive patients with schizophrenia. Compr. Psychiatry.

[16] Hunt G.E., Large M.M., Cleary M., Lai H.M.X., Saunders J.B. (2018). Prevalence of comorbid substance use in schizophrenia spectrum disorders in community and clinical settings, 1990–2017: Systematic review and meta-analysis. Drug Alcohol Depend..

[17] Kranzler H.R. (2023). Overview of Alcohol Use Disorder. Am. J. Psychiatry.

[18] Ross L.J., Wilson M., Banks M., Rezannah F., Daglish M. (2012). Prevalence of malnutrition and nutritional risk factors in patients undergoing alcohol and drug treatment. Nutrition.

[19] Butts M., Sundaram V.L., Murughiyan U., Borthakur A., Singh S. (2023). The Influence of Alcohol Consumption on Intestinal Nutrient Absorption: A Comprehensive Review. Nutrients.

[20] Kale A., Naphade N., Sapkale S., Kamaraju M., Pillai A., Joshi S., Mahadik S. (2010). Reduced folic acid, vitamin B12 and docosahexaenoic acid and increased homocysteine and cortisol in never-medicated schizophrenia patients: Implications for altered one-carbon metabolism. Psychiatry Res..

[21] Krebs M.O., Bellon A., Mainguy G., Jay T.M., Frieling H. (2009). One-carbon metabolism and schizophrenia: Current challenges and future directions. Trends Mol. Med..

[22] Raghubeer S., Matsha T.E. (2021). Methylenetetrahydrofolate (MTHFR), the One-Carbon Cycle, and Cardiovascular Risks. Nutrients.

[23] Savitz J. (2020). The kynurenine pathway: A finger in every pie. Mol. Psychiatry.

[24] Wells J.C., Sawaya A.L., Wibaek R., Mwangome M., Poullas M.S., Yajnik C.S., Demaio A. (2020). The double burden of malnutrition: Aetiological pathways and consequences for health. Lancet.

[25] Hoffman D.J., Powell T.L., Barrett E.S., Hardy D.B. (2021). Developmental origins of metabolic diseases. Physiol. Rev..

[26] Nobs S.P., Zmora N., Elinav E. (2020). Nutrition Regulates Innate Immunity in Health and Disease. Annu. Rev. Nutr..

[27] Fang Y., Xia J., Lian Y., Zhang M., Kang Y., Zhao Z., Wang L., Yin P., Wang Z., Ye C. (2023). The burden of cardiovascular disease attributable to dietary risk factors in the provinces of China, 2002–2018: A nationwide population-based study. Lancet Reg. Health-West. Pac..

[28] Popkin B.M., Corvalan C., Grummer-Strawn L.M. (2020). Dynamics of the double burden of malnutrition and the changing nutrition reality. Lancet.

[29] Lin D., Kim H., Wada K., Aboumrad M., Powell E., Zwain G., Benson C., Near A. (2022). Unemployment, homelessness, and other societal outcomes in patients with schizophrenia: A real-world retrospective cohort study of the United States Veterans Health Administration database: Societal burden of schizophrenia among US veterans. BMC Psychiatry.

[30] Li Y., Hou C.-L., Ma X.-R., Zhong B.-L., Zang Y., Jia F.-J., Lin Y.-Q., Lai K.-Y., Chiu H.-F., Ungvari G.-S. (2017). Quality of life in Chinese patients with schizophrenia treated in primary care. Psychiatry Res..

[31] Dicken S.J., Batterham R.L. (2022). Ultra-processed food: A global problem requiring a global solution. Lancet Diabetes Endocrinol..

[32] Gutwinski S., Schreiter S., Deutscher K., Fazel S. (2021). The prevalence of mental disorders among homeless people in high-income countries: An updated systematic review and meta-regression analysis. PLoS Med..

[33] Mozaffarian D., Blanck H.M., Garfield K.M., Wassung A., Petersen R. (2022). A Food is Medicine approach to achieve nutrition security and improve health. Nat. Med..

[34] McCutcheon R.A., Reis Marques T., Howes O.D. (2020). Schizophrenia—An Overview. JAMA Psychiatry.

[35] Garrido-Torres N., Rocha-Gonzalez I., Alameda L., Rodriguez-Gangoso A., Vilches A., Canal-Rivero M., Crespo-Facorro B., Ruiz-Veguilla M. (2021). Metabolic syndrome in antipsychotic-naïve patients with first-episode psychosis: A systematic review and meta-analysis. Psychol. Med..

[36] de Beaurepaire R. (2021). Binge Eating Disorders in Antipsychotic-Treated Patients With Schizophrenia: Prevalence, Antipsychotic Specificities, and Changes Over Time. J. Clin. Psychopharmacol..

[37] Benarroch L., Kowalchuk C., Wilson V., Teo C., Guenette M., Chintoh A., Nesarajah Y., Taylor V., Selby P., Fletcher P. (2016). Atypical antipsychotics and effects on feeding: From mice to men. Psychopharmacology.

[38] Cernea S., Dima L., Correll C.U., Manu P. (2020). Pharmacological Management of Glucose Dysregulation in Patients Treated with Second-Generation Antipsychotics. Drugs.

[39] Bretler T., Weisberg H., Koren O., Neuman H. (2019). The effects of antipsychotic medications on microbiome and weight gain in children and adolescents. BMC Med..

[40] Blouin M., Tremblay A., Jalbert M.-E., Venables H., Bouchard R.H., Roy M.A., Alméras N. (2008). Adiposity and Eating Behaviors in Patients Under Second Generation Antipsychotics. Obesity.

[41] Mukherjee S., Skrede S., Milbank E., Andriantsitohaina R., López M., Fernø J. (2022). Understanding the Effects of Antipsychotics on Appetite Control. Front. Nutr..

[42] Kouidrat Y., Amad A., Lalau J.-D., Loas G. (2014). Eating Disorders in Schizophrenia: Implications for Research and Management. Schizophr. Res. Treat..

[43] Braga R.J., Reynolds G.P., Siris S.G. (2013). Anxiety comorbidity in schizophrenia. Psychiatry Res..

[44] Li W., Yang Y., An F.-R., Zhang L., Ungvari G.S., Jackson T., Yuan Z., Xiang Y.-T. (2020). Prevalence of comorbid depression in schizophrenia: A meta-analysis of observational studies. J. Affect. Disord..

[45] Dakanalis A., Mentzelou M., Papadopoulou S.K., Papandreou D., Spanoudaki M., Vasios G.K., Pavlidou E., Mantzorou M., Giaginis C. (2023). The Association of Emotional Eating with Overweight/Obesity, Depression, Anxiety/Stress, and Dietary Patterns: A Review of the Current Clinical Evidence. Nutrients.

[46] McCutcheon R.A., Keefe R.S.E., McGuire P.K. (2023). Cognitive impairment in schizophrenia: Aetiology, pathophysiology, and treatment. Mol. Psychiatry.

[47] Chan R.C.K., Wang L., Lui S.S.Y. (2022). Theories and models of negative symptoms in schizophrenia and clinical implications. Nat. Rev. Psychol..

[48] Wang L.L., Tam M.H.W., Ho K.K.Y., Hung K.S.Y., Wong J.O.Y., Lui S.S.Y., Chan R.C.K. (2023). Bridge centrality network structure of negative symptoms in people with schizophrenia. Eur. Arch. Psychiatry Clin. Neurosci..

[49] Kulkarni D.P., Kamath V.D., Stewart J.T. (2017). Swallowing Disorders in Schizophrenia. Dysphagia.

[50] Thornicroft G., Sunkel C., Alikhon Aliev A., Baker S., Brohan E., El Chammay R., Davies K., Demissie M., Duncan J., Fekadu W. (2022). The Lancet Commission on ending stigma and discrimination in mental health. Lancet.

[51] Domany Y., Weiser M. (2020). Insights into metabolic dysregulations associated with antipsychotics. Lancet Psychiatry.

[52] Pillinger T., McCutcheon R.A., Vano L., Mizuno Y., Arumuham A., Hindley G., Beck K., Natesan S., Efthimiou O., Cipriani A. (2020). Comparative effects of 18 antipsychotics on metabolic function in patients with schizophrenia, predictors of metabolic dysregulation, and association with psychopathology: A systematic review and network meta-analysis. Lancet Psychiatry.

[53] Jamilian H., Solhi H., Jamilian M. (2014). Randomized, Placebo-Controlled Clinical Trial of Omega-3 as Supplemental Treatment in Schizophrenia. Glob. J. Health Sci..

[54] Agostoni C., Nobile M., Ciappolino V., Delvecchio G., Tesei A., Turolo S., Crippa A., Mazzocchi A., Altamura C.A., Brambilla P. (2017). The Role of Omega-3 Fatty Acids in Developmental Psychopathology: A Systematic Review on Early Psychosis, Autism, and ADHD. Int. J. Mol. Sci..

[55] Ghaderi A., Banafshe H.R., Mirhosseini N., Moradi M., Karimi M.A., Mehrzad F., Bahmani F., Asemi Z. (2019). Clinical and metabolic response to vitamin D plus probiotic in schizophrenia patients. BMC Psychiatry.

[56] Wyatt R.J., Vaughan T., Galanter M., Kaplan J., Green R. (1972). Behavioral Changes of Chronic Schizophrenic Patients Given L-5-Hydroxytryptophan. Science.

[57] Rastogi R.B., Singhal R.L., Lapierre Y.D. (1981). Effects of short- and long-term neuroleptic treatment on brain serotonin synthesis and turnover: Focus on the serotonin hypothesis of schizophrenia. Life Sci..

[58] Ghaderi A., Banafshe H.R., Mirhosseini N., Moradi M., Karimi M.A., Mehrzad F., Bahmani F., Asemi Z. (1992). Effect of a Low-Tryptophan Diet as an Adjuvant to Conventional Neuroleptic Therapy in Schizophrenia. Clin. Neuropharmacol..

[59] Hare S.M., Adhikari B.M., Mo C., Chen S., Wijtenburg S.A., Seneviratne C., Kane-Gerard S., Sathyasaikumar K.V., Notarangelo F.M., Schwarcz R. (2023). Tryptophan challenge in individuals with schizophrenia and healthy controls: Acute effects on circulating kynurenine and kynurenic acid, cognition and cerebral blood flow. Neuropsychopharmacology.

[60] McCreadie R.G., Kelly C., Connolly M., Williams S., Baxter G., Lean M., Paterson J.R. (2005). Dietary improvement in people with schizophrenia: Randomised controlled trial. Br. J. Psychiatry.

[61] Newcomer J.W. (2007). Metabolic considerations in the use of antipsychotic medications: A review of recent evidence. J. Clin. Psychiatry.

[62] Huang J., Kang D., Zhang F., Yang Y., Liu C., Xiao J., Long Y., Lang B., Peng X., Wang W. (2022). Probiotics Plus Dietary Fiber Supplements Attenuate Olanzapine-Induced Weight Gain in Drug-Naïve First-Episode Schizophrenia Patients: Two Randomized Clinical Trials. Schizophr. Bull..

[63] Osmond H., Hoffer A. (1962). Massive Niacin Treatment in Schizophrenia. Lancet.

[64] Pitt B., Pollitt N. (1971). Ascorbic Acid and Chronic Schizophrenia. Br. J. Psychiatry.

[65] Vaughan K., McConaghy N. (1999). Megavitamin and Dietary Treatment in Schizophrenia: A Randomised, Controlled Trial. Aust. N. Z. J. Psychiatry.

[66] Peña-Romero A.C., Navas-Carrillo D., Marín F., Orenes-Piñero E. (2018). The future of nutrition: Nutrigenomics and nutrigenetics in obesity and cardiovascular diseases. Crit. Rev. Food Sci. Nutr..

[67] Marcum J.A. (2020). Nutrigenetics/Nutrigenomics, Personalized Nutrition, and Precision Healthcare. Curr. Nutr. Rep..

[68] Wuni R., Kuhnle G.G.C., Wynn-Jones A.A., Vimaleswaran K.S. (2022). A Nutrigenetic Update on CETP Gene–Diet Interactions on Lipid-Related Outcomes. Curr. Atheroscler. Rep..

[69] Usategui-Martín R., De Luis-Román D.-A., Fernández-Gómez J.M., Ruiz-Mambrilla M., Pérez-Castrillón J.-L. (2022). Vitamin D Receptor (VDR) Gene Polymorphisms Modify the Response to Vitamin D Supplementation: A Systematic Review and Meta-Analysis. Nutrients.

[70] Cornelis M.C., Byrne E.M., Esko T., Nalls M.A., Ganna A., Paynter N., Monda K.L., Amin N., Fischer K., The Coffee and Caffeine Genetics Consortium (2015). Genome-wide meta-analysis identifies six novel loci associated with habitual coffee consumption. Mol. Psychiatry.

[71] Fujimaki M., Saiki S., Li Y., Kaga N., Taka H., Hatano T., Ishikawa K.I., Oji Y., Mori A., Okuzumi A. (2018). Serum caffeine and metabolites are reliable biomarkers of early Parkinson disease. Neurology.

[72] Takeshige-Amano H., Saiki S., Fujimaki M., Ueno S.I., Li Y., Hatano T., Ishikawa K.I., Oji Y., Mori A., Okuzumi A. (2020). Shared Metabolic Profile of Caffeine in Parkinsonian Disorders. Mov. Disord..

[73] Trubetskoy V., Pardiñas A.F., Qi T., Panagiotaropoulou G., Awasthi S., Bigdeli T.B., Bryois J., Chen C.Y., Dennison C.A., Hall L.S. (2022). Mapping genomic loci implicates genes and synaptic biology in schizophrenia. Nature.

[74] Hunjan A.K., Hübel C., Lin Y., Eley T.C., Breen G. (2021). Association between polygenic propensity for psychiatric disorders and nutrient intake. Commun. Biol..

[75] Mullins V.A., Bresette W., Johnstone L., Hallmark B., Chilton F.H. (2020). Genomics in Personalized Nutrition: Can You “Eat for Your Genes”?. Nutrients.

[76] Blokland G.A.M., Grove J., Chen C.-Y., Cotsapas C., Tobet S., Handa R., Ripke S., Neale B.M., Corvin A., Walters J.T. (2022). Sex-Dependent Shared and Nonshared Genetic Architecture Across Mood and Psychotic Disorders. Biol. Psychiatry.

[77] Cervenka I., Agudelo L.Z., Ruas J.L. (2017). Kynurenines: Tryptophan’s metabolites in exercise, inflammation, and mental health. Science.

[78] Maruvada P., Lampe J.W., Wishart D.S., Barupal D., Chester D.N., Dodd D., Djoumbou-Feunang Y., Dorrestein P.C., Dragsted L.O., Draper J. (2020). Perspective: Dietary Biomarkers of Intake and Exposure—Exploration with Omics Approaches. Adv. Nutr..

[79] Voruganti V.S. (2023). Precision Nutrition: Recent Advances in Obesity. Physiology.

[80] Samieri C., Yassine H.N., Melo van Lent D., Lefèvre-Arbogast S., van de Rest O., Bowman G.L., Scarmeas N. (2022). Personalized nutrition for dementia prevention. Alzheimer’s Dement..

[81] Davidson M., Rashidi N., Nurgali K., Apostolopoulos V. (2022). The Role of Tryptophan Metabolites in Neuropsychiatric Disorders. Int. J. Mol. Sci..

[82] Kuuskmäe C., Philips M.A., Kilk K., Haring L., Kangro R., Seppo I., Zilmer M., Vasar E. (2023). Kynurenine pathway dynamics in patients with schizophrenia spectrum disorders across the disease trajectory. Psychiatry Res..

[83] Wang K.C., Chang H.Y. (2018). Epigenomics: Technologies and Applications. Circ. Res..

[84] Stover P.J., James W.P.T., Krook A., Garza C. (2018). Emerging concepts on the role of epigenetics in the relationships between nutrition and health. J. Intern. Med..

[85] Ma Y., Ordovas J.M. (2017). The integration of epigenetics and genetics in nutrition research for CVD risk factors. Proc. Nutr. Soc..

[86] Marx W., Moseley G., Berk M., Jacka F. (2017). Nutritional psychiatry: The present state of the evidence. Proc. Nutr. Soc..

[87] Jacka F.N. (2017). Nutritional Psychiatry: Where to Next?. EBioMedicine.

[88] Lindsay K.L., Buss C., Wadhwa P.D., Entringer S. (2019). The Interplay Between Nutrition and Stress in Pregnancy: Implications for Fetal Programming of Brain Development. Biol. Psychiatry.

[89] Susser E.S. (1992). Schizophrenia After Prenatal Exposure to the Dutch Hunger Winter of 1944–1945. Arch. Gen. Psychiatry.

[90] St Clair D. (2005). Rates of Adult Schizophrenia Following Prenatal Exposure to the Chinese Famine of 1959–1961. JAMA.

[91] Sullivan S., Wills A., Lawlor D., McGrath J., Zammit S. (2013). Prenatal vitamin D status and risk of psychotic experiences at age 18 years—A longitudinal birth cohort. Schizophr. Res..

[92] Paquin V., Lapierre M., Veru F., King S. (2021). Early Environmental Upheaval and the Risk for Schizophrenia. Annu. Rev. Clin. Psychol..

[93] Cortés-Albornoz M.C., García-Guáqueta D.P., Velez-van-Meerbeke A., Talero-Gutiérrez C. (2021). Maternal Nutrition and Neurodevelopment: A Scoping Review. Nutrients.

[94] Lieberman J.A., Small S.A., Girgis R.R. (2019). Early Detection and Preventive Intervention in Schizophrenia: From Fantasy to Reality. Am. J. Psychiatry.

[95] Deng J., Zhu X., Chen Z., Fan C.H., Kwan H.S., Wong C.H., Shek K.Y., Zuo Z., Lam T.N. (2017). A Review of Food–Drug Interactions on Oral Drug Absorption. Drugs.

[96] Xu Y., Amdanee N., Zhang X. (2021). Antipsychotic-Induced Constipation: A Review of the Pathogenesis, Clinical Diagnosis, and Treatment. CNS Drugs.

[97] Matrisciano F. (2023). Functional Nutrition as Integrated Intervention for In- and Outpatientwith Schizophrenia. Curr. Neuropharmacol..

[98] Tsai M.-T., Chang T.-H., Wu B.-J. (2018). Prognostic impact of nutritional risk assessment in patients with chronic schizophrenia. Schizophr. Res..

